# A dynamic estimation of the daily cumulative cases during infectious disease surveillance: application to dengue fever

**DOI:** 10.1186/1471-2334-10-136

**Published:** 2010-05-27

**Authors:** Pei-Hung Chuang, Jen-Hsiang Chuang, I-Feng Lin

**Affiliations:** 1 Institute of Public Health, School of Medicine, National Yang-Ming University, Taipei, Taiwan; 2 Epidemic Intelligence Center, Centers for Disease Control, Taipei, Taiwan; 3 Institute of Environmental & Occupational Health Sciences, National Yang-Ming University, Taipei, Taiwan; 4 Institute of Biomedical Informatics, School of Medicine, National Yang-Ming University, Taipei, Taiwan

## Abstract

**Background:**

In infectious disease surveillance, when the laboratory confirmation of the cases is time-consuming, there is often a time lag between the number of suspect cases and the number of confirmed cases. This study proposes a dynamic statistical model to estimate the daily number of new cases and the daily cumulative number of infected cases, which was then applied to historic dengue fever data.

**Methods:**

The duration between the date of disease onset and date of laboratory confirmation was assumed to follow a gamma distribution or a nonparametric distribution. A conditional probability of a case being a real case among the unconfirmed cases on a given date was then calculated. This probability along with the observed confirmed cases was integrated to estimate the daily number of new cases and the cumulative number of infected cases.

**Results:**

The distribution of the onset-to-confirmation time for the positive cases was different from that of the negative cases. The daily new cases and cumulative epidemic curves estimated by the proposed method have a lower absolute relative bias than the values estimated solely based on the available daily-confirmed cases.

**Conclusion:**

The proposed method provides a more accurate real-time estimation of the daily new cases and daily cumulative number of infected cases. The model makes use of the most recent "moving window" of information relative to suspect cases and dynamically updates the parameters. The proposed method will be useful for the real-time evaluation of a disease outbreak when case classification requires a time-consuming laboratory process to identify a confirmed case.

## Background

Timeliness and accuracy of case reporting are two of the most important performance measures when evaluating an infectious disease surveillance system [1-5]. Laboratory confirmation is usually needed for case diagnosis in many infectious diseases. When laboratory confirmation of the diagnosis is time-consuming, however, there is often a time-lag between the onset date of symptoms and the diagnosis date [6]. For example, the median time for confirmation of the meningococcal disease is about 13 days based on the National Notifiable Diseases Surveillance System (NNDSS) dataset for the United States from 1999 to 2001 [7]. Time from disease onset to diagnosis has been also reported to account for most of the delay in case reporting in Korea [8]. Correct estimation of daily cases or daily-cumulative infected cases helps the implement of immediate disease control and allows prevention activities for infectious diseases to be instituted [6]. Using a disease surveillance system, one is able to apply statistical methods, such as cumulative sum (CuSum) [9,10] or autoregressive integrated moving average (ARIMA) [11-15], in order to forecast an epidemic curve or to detect aberrations in disease spread. These estimations are based on having a correct daily number of cases or a daily-cumulative number of cases.

An epidemic of dengue fever occurs every year in southern Taiwan. In the year 2002 in particular, there was a large-scale epidemic with 5,388 confirmed cases out of totally 15,221 suspect cases [16]. This epidemic continued until March 2003. Surveillance and the control of dengue fever have become one of the most important routine areas of work at the Taiwan Centers for Disease Control (Taiwan CDC) in recent years. By 2008, Taiwan CDC had defined a confirmed case of dengue fever as an acute febrile illness together with one of the following criteria: (1) isolation of dengue virus; (2) demonstration of positive results by real-time reverse transcription--polymerase chain reaction (real-time RT-PCR); (3) demonstration of positive seroconversion or a fourfold increase in dengue-specific IgM or IgG antibody titers in paired serum samples; or (4) demonstration of high-titer dengue-specific IgM and IgG antibodies in a single serum specimen [17-19]. When the dengue fever case classification only included confirmed cases by this protocol, the time needed for isolating the agent or measuring the titers for the acute- and convalescent-phase serum specimens was significant. The result was that there was a gap between the available daily cases or the daily-cumulative cases for given a day and the actual final confirmed cases for the same day given that all diagnosis had been completed on that given day.

Assuming that a time cost for laboratory confirmation of diagnosis is sometimes inevitable, daily numbers of infected cases and daily-cumulative number of infected cases may be underestimated during an epidemic. The objective of this study was to develop a new method to estimate the number of daily cumulative cases and that this method will be applied to dengue fever in Taiwan, as an example.

## Methods

### Data sources

Since there are almost no dengue fever cases occurred during the winter in Taiwan, we chose May 1 as the beginning of the dengue epidemic season when estimating the cumulative epidemic curve. The data come from the dengue notification dataset containing suspect cases in Taiwan whose date of onset was from May 1, 2005 to April 30, 2007. All serum samples from suspect cases were sent to the two reference laboratories at the Taiwan CDC in order to further identify if they were positive (dengue fever infected) or negative cases. The reason we retrieved data based on the date of onset rather than the report date was to avoid the influence of lag reporting on the course of the disease. All imported cases of the disease were removed. The variables we used were the date of onset, the date of laboratory confirmation (diagnosis date), and the final confirmed status of each suspect case (a binary variable that is either positive or negative). In this article, we use confirmed dengue cases and positive cases interchangeably. No personal identification information was contained in the dataset.

**Figure 1:**
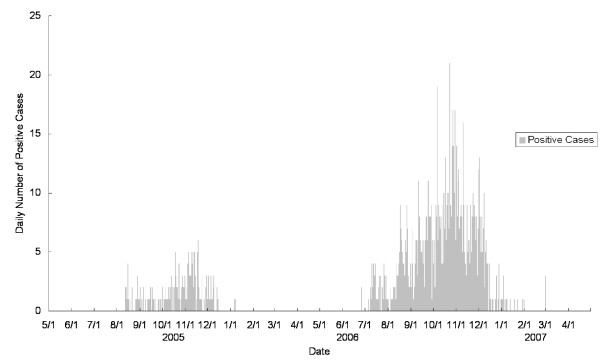
**The daily number of dengue new cases during the 2006-2007 season based on their final status**.

There were 515 positive cases out of 841 local suspect cases during the 2005-2006 season and 1,092 positive cases out of 2,360 local suspect cases during the 2006-2007 season. The median values (interquartile-range; IQR) of the onset-to-confirmation time (OC-time) for positive and negative cases were 9.5 (11) and 20 (14) days respectively for the 2005-2006 season. The median values (IQR) of the OC-time for positive and negative cases were 7 (9) and 18 (7) days respectively for the 2006-2007 season. The OC-time for positive cases was, in general, shorter than that for the negative cases. The standard deviations of the OC-time for the positive cases and negative cases were also different. Figure 1 shows that the epidemic started in the late June of 2006, and had a peak around October to November, then had nearly vanished by February 2007, based on the final status of each case.

### The proposed method

#### Confirmation status of the suspect cases

Our proposed method estimates the real-time daily new number of cases and the daily cumulative number of dengue cases; specifically, these numbers of dengue cases are updated daily. Let *c *be the "current" date when the number of dengue cases is to be estimated. In this study, the date *c *runs from May 1, 2006 to April 30, 2007. For the *i*^th ^reported suspect case counting from the 1^st ^day of the epidemic season, that is May 1 in this study, we define the suspect case's onset date as *O*_*i *_and the laboratory confirmation date as *D*_*i *_If *D*_*i *_>*c *on date *c*, the case *i *does not have a confirmation result as of date *c*; on the other hand, if *D*_*i *_≤ *c*, this case *i *is either confirmed to be a positive dengue case or has a negative result as of date *c*. Let the final confirmation status for the *i*^th ^suspect case be, where as a positive dengue case, and as a negative case. In the situation where there are unconfirmed suspect cases as of date *c*, we assigned a probability of being a dengue case, *P*(*i*), to those unconfirmed cases (*D *≤ *c*). Then for each suspect case *i*, the expected final confirmation status on date *c*, *E*_*i*_(*c*), can be written as(1)

The values of *P*_*i*_(*c*), and *E*_*i*_(*c*)are updated for each case *i *every day. Without applying the proposed method, one would be only able to observe the case status from the upper part of *E*_*i*_(*c*)in equation (1). After *E*_*i*_(*c*)is calculated for each suspect case, daily new cases are easily estimated by summing the *E*_*i*_(*c*)over all new suspect cases on date *c*, and cumulative cases can be obtained by summing *E*_*i*_(*c*)over all cases from *i *= 1 to the newest suspect cases on date *c*.

#### Estimation of the probability of being dengue case, *P*_*i*_(*c*) among unconfirmed suspect cases

*P*_*i*_(*c*)is estimated for unconfirmed cases using information from the confirmed cases before date *c *within one year. Let *T*_*i *_be the onset-to-confirmation time (OC-time), the time interval between the onset date and the lab-confirmation date. The OC-time for the *i*^th ^suspect case as of date *c*, *t*_*i*_(*c*), is calculated as follows,(2)

The *t*_*i*_(*c*) is the OC-time for confirmed cases and the censored OC-time for unconfirmed cases on date *c*.

By applying several steps of Bayes' rules, the probability *P*_*i*_(*c*) is given by:(3)

To estimate *P*_*i*_(*c*) using the information as of date *c*, we applied the following steps. We first estimated *P*(*Y*_*i *_= 1) by calculating the proportion of confirmed positive dengue cases out of the suspect cases using the data with onset date within 1 year before the date *c*. Based on a parametric approach, we assumed the OC-time for a given case status, *P*(*T*|*y*_*i*_), follow a gamma distribution. Gamma distributions are frequently used to fit time-delay distributions or time event distributions when carrying out disease surveillance analysis [20,21]. The probability density function of the gamma distribution is , where . The gamma distribution is denoted by with two parameters, the shape parameter *α *and the scale parameter *β*, and the mean and variance are *αβ *and *αβ*^2^, respectively. The values of *α *and *β *were estimated and solved by setting up the sample mean and the sample variance of the OC-time equal to *αβ *and *αβ*^2^, respectively. As mentioned in the previous section, the mean and standard deviation of the OC-time were different between positive and negative cases, we estimated different sets of *α *and *β *for the positive dengue cases (Y = 1) and negative cases (Y = 0) separately. A nonparametric approach was also performed in which the probability *P*(*T *>*t*_*i*_(*c*)|*Y*_*i*_) was simply replaced with the cumulative proportion of confirmed data given their final status. Both the parametric and nonparametric models were based on the data within a 1-year "moving window" before date *c*. The *P*_*i*_*(c) *and *E*_*i*_*(c) *were also updated everyday.

### Evaluation of the proposed methods

To evaluate the performance of the proposed method, we estimated the daily new cases and daily cumulative cases for each calendar date *c *from May 1, 2006 to April 30, 2007. Four epidemic curves are presented. There are:

(1) The final status curve, which is the number of dengue cases based on their final confirmation status ("Real data", "gold standard").

(2) The daily confirmed curve, which is the number of dengue cases based on the confirmed cases as of date *c*.

(3) The gamma-model curve, which is the number of dengue cases, estimated using the gamma distribution.

(4) The nonparametric-model curve, which is the number of dengue cases, estimated using the nonparametric distribution.

To summarize the magnitude of the bias, we defined the absolute relative bias (ARB) at date *c *as: 

where  are the cumulative cases estimated by the proposed methods or by the confirmed cases observed on date *c *without using the proposed methods, *N*_*c *_and are the cumulative confirmed cases based on the final status ("real data", "gold standard"). An ARB closer to zero is a more accurate estimate.

All analyses were performed using SAS 9.1.3 software (SAS Institute, Inc., Cary, NC). Special SAS macros for estimating the cumulative cases and daily new cases, based on our proposed model, were developed.

## Results

**Figure 2:**
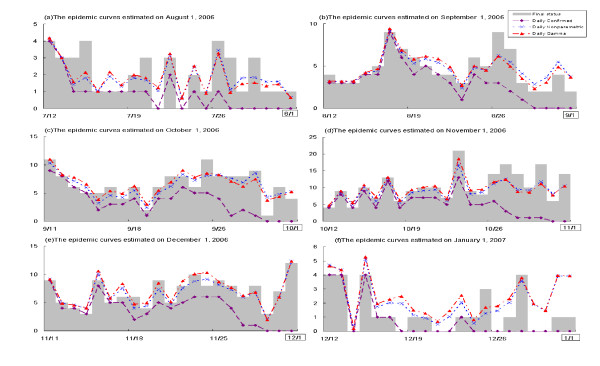
**Dynamic epidemic curves (daily new cases) within 3 weeks before the view date**. (a) The epidemic curves estimated on August 1^st^, 2006. (b) The epidemic curves estimated on September 1^st^, 2006. (c) The epidemic curves estimated on October 1^st^, 2006. (d) The epidemic curves estimated on November 1^st^, 2006. (e) The epidemic curves estimated on December 1^st^, 2006. (f) The epidemic curves estimated on January 1^st^, 2007. Each point of the curves was calculated as the date changed. See text for details.

Figure 2 compares the daily *new *cases estimated by the proposed models, the confirmed curve (confirmed new cases observed on date *c *without using the proposed models), and the final status curve (confirmed new cases based on final status; the "gold standard"). Since the daily *new *cases estimated by the proposed methods or the daily new cases observed on date *c *were different when viewed on different dates, arbitrary view dates of August 1, 2006 (beginning of the epidemic), September 1, 2006 (rising stage, before the peak), October 1, 2006 (rising stage, before but closer the peak) and November 1, 2006 (around the peak), December 1, 2006 (downward stage, after the peak), and January 1, 2007 (end of the epidemic) were chosen to illustrate the results of the estimated daily *new *cases. Each graph in Figure 2 shows the epidemic curves three weeks before the view dates. When viewed on August 1, most of suspect cases had been lab-confirmed before July 15 thus all four curves were close to each others before that date. From July 19 to August 1, the estimated curves by the proposed methods (red dashed lines with triangle symbols by gamma distribution and blue dashed lines with cross symbols by the nonparametric method) were much closer to the final status curve (shaded area) than that by simply observing the daily-confirmed new cases (purple dashed line). Similar patterns were observed when the results are viewed on September 1, October 1, November 1, and December 1. The observed daily-confirmed cases usually underestimated the true daily new cases as would be expected, especially within the two weeks before the view date. The curves estimated by gamma distribution or the nonparametric approach were quite similar. However, the daily new cases, as estimated by the proposed method, did not give an accurate estimate towards the end stage of the epidemic, namely when viewed on January 1, 2007.

**Figure 3:**
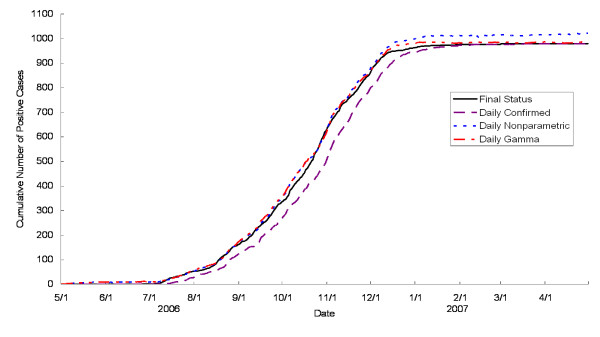
**Dynamic cumulative epidemic curves**. Each point of curves was calculated as the date changed.

The epidemic curves in terms of daily *cumulative *cases are shown on Figure 3. In this figure, the cumulative number of positive cases was updated every day. The two estimated daily-cumulative curves by the proposed methods are quite similar to the final status curve before January but again the proposed method does not work well during the end stage of the epidemic. Table 1 compares the ARB of the daily cumulative number of positive cases between the different methods. After the first confirmed positive case appeared on July 6, 2006, the estimates based on the gamma model results in an estimate closer to the real data than the other methods. For other two curves, the nonparametric method performs worst at the end of the epidemic after January 1 and there was about 20 cases higher than the final status curve. The daily confirmed curve was about 50 cases lower than the final status curve during the peak of epidemic.

**Table 1:** Comparisons of the different methods for estimating the daily cumulative dengue cases by absolute relative bias during the 2006-2007 season

			Absolute Relative Bias†					
			
			Gamma		Nonparametric		Daily-confirmed	
Year	Date	Positive cases	Median	(IQR)	Median	(IQR)	Median	(IQR)
2006	Jun. 1~Jul. 5	3	440.3%	(57.8%)	363.6%	(50.5%)	100.0%	(0%)
	Jul. 6*~Jul. 31	50	5.7%	(13.2%)	8.5%	(10.9%)	67.7%	(19.1%)
	Aug. 1~Aug. 31	108	5.8%	(11.2%)	5.3%	(8.7%)	30.1%	(9.8%)
	Sep. 1~Sep. 30	173	6.9%	(2.2%)	4.0%	(1.7%)	21.7%	(3.0%)
	Oct. 1~Oct. 31	291	6.7%	(5.6%)	5.9%	(5.8%)	17.3%	(2.9%)
	Nov. 1~Nov. 30	230	1.5%	(1.2%)	2.3%	(1.3%)	11.9%	(6.0%)
	Dec. 1~Dec. 31	108	1.7%	(1.5%)	3.0%	(1.9%)	4.7%	(5.3%)
2007	Jan. 1~Jan. 31	13	1.4%	(0.6%)	3.9%	(0.3%)	0.8%	(0.8%)
	Feb. 1~Feb. 28	0	0.9%	(0.2%)	3.7%	(0.2%)	-	
	Mar. 1~Mar. 31	3	0.5%	(0.3%)	3.5%	(0.4%)	0.1%	(0.2%)
	Apr. 1~Apr. 30	0	0.6%	(0.3%)	3.9%	(0.3%)	-	

* The first confirmed positive dengue case appeared on July 6, 2006.

^† ^Absolute relative bias (ARB) is calculated by the absolute value of the differences between the estimated and the expected daily cumulative cases divided by the expected daily cumulative cases. See text for details. An ARB closer to zero is a more accurate estimate.

**Figure 4:**
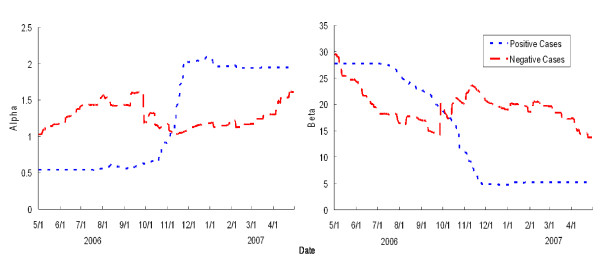
**Parameter estimates of gamma distribution used in calculating the daily case counts**. (a) The shape parameter *α *of gamma distribution. (b) The scale parameter *β *of gamma distribution. Each point of curves was calculated as the date changed.

Figure 4 showed the daily parameter estimates, *α *and *β*, of the gamma distributions used to dynamically calculate the daily number of positive cases. The parameter estimates varied from day to day and thus the probability of being a positive case changes. For the negative cases, the parameters had a jump during late September.

## Discussion

As noted previously, timeliness and accuracy are the two of most important characteristics when we evaluate an infectious disease surveillance system. Our results show that when an infectious disease required a time-consuming process for diagnosis, such as the dengue fever using the previously mentioned protocol, the actual daily number of infected cases and cumulative positive cases are potentially underestimated. The proposed method dynamically updates the parameters daily by making use of the most recently available information on suspect cases, and then performed estimates with a lower absolute relative bias than when using observed daily lab-confirmed cases only. As shown in Table 1, the proposed method performed a lower median absolute relative bias (ABS range 1.7% ~ 8.5%) than those solely based on daily confirmed cases (ABS range 4.7% ~ 67.7%) between July 6 and December 31. These dates covered the rising stage and around the peak stage which were of public health interest. The proposed method provides a more accurate estimate of the epidemic curves when applied to the dengue fever dataset for Taiwan during the 2006-2007 season. Based on these results, this approach can be used for the real-time evaluation of the severity of a disease outbreak when case classification requires that a confirmed case involves a time-consuming process.

In this study, we first established the different distributions for the onset-to-confirmation time of the positive cases and negative cases. Next, either a gamma distribution was assumed in order to estimate the probability of being a confirmed case given cases status in equation (1), or, alternatively, a nonparametric approach was used. We actually experimented with several types of distribution. The estimates using a log-normal distribution were numerically very similar to the results for the gamma distribution. The estimates using a Weibull distribution did not perform as well as the gamma distribution applied in our dengue fever data. From Figure 4, we learn that the shape parameter *α *changed from 0.5 to 2 and therefore an exponential distribution may not be appropriate. For simplification, we have chosen to present only the results from the gamma distribution as one example of a parametric approach and compare this with a nonparametric approach. As shown in Figure 2 for daily new cases, the differences in the estimates based on parametric approach with Gamma distribution and those with nonparametric approaches were minor. The Figure 3 and Table 1 for cumulative cases showed that a gamma distribution is a more appropriate assumption for the onset-to-diagnosis time when estimating the probability of being a positive case using the dengue fever example; nonetheless, the difference between the gamma and the nonparametric method is again only slight except towards the end stage of the epidemic after January 1. The reason that the nonparametric method did not work well after January 1 is because *P*(*Y*_*i *_= 1), *P*(*Y*_*i *_= 0), and *P*(*T*|*y*_*i*_) had not changed substantially, resulting in a near constant estimate of the daily positive cases.

In practice, any form of the probability of being a positive case can be assumed. It is also not restricted to certain distributions when the models are adapted to different types of infectious disease. When applying this approach to other diseases, researchers should investigate several distributions according to the shape of their data and choose an appropriate one based on some appropriate measures, for instance, those shown in Table 1.

Our method estimated the probability of being a positive case based on the data within a 1-year "moving window" before date *c *and updated *P*_*i*_*(c) *and *E*_*i*_*(c) *everyday. The epidemic profiles of dengue fever are different from one year to another in Taiwan. Choosing the data from most recent one year was done in order to insure that there was enough information to cover a whole epidemic season. In the early stage of the 2006-2007 season, the data from the 2005-2006 season actually contributed more to estimating the daily cases counts. In this study, even the epidemic profiles were not necessarily the same between the 2005-2006 and 2006-2007 seasons, the proposed methods performed well.

The study shows that before the first positive case appeared on July 6, the proposed method did not work well and are not that useful (Table 1). Our method worked well after the first positive case appeared during the 2006-2007 season. Indeed, it needed only four days to be able to consistently estimate the final status curve. In the 2006-2007 season, Taiwan CDC activated a central command center for intensively dengue epidemic control on October 2. The task of this command center included expanded blood sample collection and it is likely that this resulted in more suspect cases for laboratory confirmation, which might have led to a lower proportion of positive cases. This would influence the estimation of probability of being a positive case over the following few days. As we can see on Figure 4, it also influenced the estimation of the parameters for negative cases. While our manuscript was being prepared, the Taiwan CDC changed their laboratory protocol for dengue fever to one that requires only a single laboratory test for dengue surveillance and control. The result is a substantial reduction in the waiting time for laboratory confirmation. However, confirmation time can never be completely avoided with dengue fever. A situation where a large number of serum specimens are sent for diagnosis at the same time will result in overloading at the laboratory, which might increase the confirmation waiting time. As described previously, the estimation used information based on a "moving window" time period before the estimated date and the parameters of the model are updated everyday. Since the observed confirmed cases counts on date *c *are always underestimated as long as there is a time lag, our method potentially can be applied while waiting for further investigation of the status of cases.

There are some limitations to our method. Firstly, the approach needs sufficient historical data to be available in order estimate the parameters of the model; therefore our model cannot be applied effectively to an emerging disease, such as SARS or avian flu. Secondly, we used confirmed cases, the dates of onset of which were within 1-year before the date estimated and if a case needs more than 1-year for diagnosis such a case might never provide any information to the parameter estimation; in such a circumstance a different "moving window" needs to be chosen. Thirdly, when missing diagnosis dates exist, the estimated curve using the nonparametric method cannot converge with the final status curve. There were 134 and 289 cases missing confirmation results for the 2005-2006 season and the 2006-2007 season, respectively, at the time that the manuscript was prepared. The nonparametric method estimates by plugging in the cumulative proportion of confirmed data given the final status. As we mention before, at the end stage of epidemic, the probabilities in equation(2) almost remained unchanged. In this study, we could only assume that the proportion of positive cases out of all suspect cases among the missing observations were similar to those having results, which basically assumes that the missing data were missing at random thus ignorable.

## Conclusion

When diagnosis of infectious diseases required laboratory confirmation, the time lag between onset and confirmation of a positive cases often exists and case counts are usually underestimated. This study has proposed a statistical method that more accurately estimates the real-time daily new cases and daily cumulative number of infected cases using a dengue fever epidemic as an example. The model makes use of the most recent "moving window" of information on suspect cases and dynamically updated the parameters of the assumed probability distributions. Different parametric or nonparametric distributions of the onset-to-confirmation times can be specified for different infectious diseases. The results show that, after the first confirmed case occurred, the estimated daily new cases or the cumulative case count fit the real data well compared to the daily counts based only on the available confirmed cases; this was done by assuming a gamma distribution for the onset to confirmation times and involved the use of a dynamic one-year "moving window" of suspected cases when applied to dengue fever outbreaks in Taiwan. This method can be used for the real-time evaluation of a disease outbreak when case diagnosis requires time-consuming laboratory process.

## Competing interests

The authors declare that they have no competing interests.

## Authors' contributions

IFL designed and conducted the study, and finalized the manuscript. PHC participated in the design of the study, performed the statistical analyses, and drafted the manuscript. JHC helped conceive the study, participated in the data collection, gave input to the manuscript, and provided medical advice from the public health perspective. All authors have read and approved the final manuscript.

## Pre-publication history

The pre-publication history for this paper can be accessed here:

http://www.biomedcentral.com/1471-2334/10/136/prepub
